# Incidence and Outcomes of Upper GI Bleeding in Hospitalized SARS-CoV-2 Patients

**DOI:** 10.1155/grp/4358786

**Published:** 2025-03-31

**Authors:** Erin Sanzone, Katherine Gheysens, Krystal Hunter, Adib Chaaya, Sangita Phadtare

**Affiliations:** ^1^Cooper Medical School of Rowan University, Camden, New Jersey, USA; ^2^Department of Gastroenterology, Virtua Gastroenterology, Cherry Hill, New Jersey, USA; ^3^Cooper Research Institute, Cooper Medical School of Rowan University, Camden, New Jersey, USA; ^4^Department of Gastroenterology, Cooper University Health Care, Camden, New Jersey, USA; ^5^Department of Biomedical Sciences, Cooper Medical School of Rowan University, Camden, New Jersey, USA

**Keywords:** duodenal ulcer, endoscopy, esophagitis, gastrointestinal bleed, SARS-CoV-2 virus

## Abstract

**Background:** In March 2020, the severe acute respiratory distress syndrome coronavirus 2 (COVID-19) became a worldwide pandemic. Recently, it has been shown that direct entry of this virus in the gastrointestinal (GI) epithelial cells causes tissue damage and the use of anticoagulants increases the risk of GI bleeding. These pose real concerns for the gastroenterologists concerning the mortality, overall incidence, and management of upper GI bleeding in SARS-CoV-2–positive patients.

**Methods:** This retrospective study includes patients 18 years or older admitted to our health system with an upper GI bleed (UGB). Patients with possible UGB, endoscopy, and SARS-CoV-2–positive testing (*n* = 587) formed the initial cohort. In-depth data were collected for symptoms, medications, source of bleeding, and interventions for subsets of test and control subjects.

**Results:** Duodenal ulcer was the most common etiology for GI bleeding in SARS-CoV-2–positive patients, while esophagitis was the most common etiology in control subjects. SARS-CoV-2–positive patients had significant progressive anemia and had to be given more blood transfusions, steroids, proton pump inhibitors, and immunosuppressants. In-hospital mortality was greater in the experimental group (12.8%) than in the control group (5.1%). Furthermore, the SARS-CoV-2–positive patients had more therapeutic interventions compared to the SARS-CoV-2–negative patients. Nearly one-quarter of all patients had an endoscopy over 48 h after bleeding was observed.

**Conclusions:** Healthcare providers should be aware of the greater therapeutic needs of SARS-CoV-2–positive patients with UGB. Our data helps shed light on the relationship between SARS-CoV-2 and GI bleeding due to SARS-CoV-2–related tissue damage and treatment affecting the GI tract.

## 1. Introduction

In March of 2020, the severe acute respiratory distress syndrome coronavirus 2 (SARS-CoV-2, COVID-19) became a worldwide pandemic. COVID-19 was initially regarded as a severe respiratory illness. As understanding of the disease evolved, its impact on other systems of the body and associated extrapulmonary complications have become more apparent. Patients suffering with the COVID-19 disease frequently experience GI-related symptoms such as nausea, vomiting, and diarrhea [1]. It has been shown that GI manifestations, especially diarrhea and GI bleeding, contribute to electrolyte abnormalities, affecting kidney health and exacerbating complications caused by the SARS-CoV-2 virus infection. This in turn contributes to morbidity and mortality in COVID-19 patients [2].

Recent evidence suggests that the mechanism underlying the entry of the SARS-CoV-2 virus into the GI epithelial cells leading to tissue damage is the same as that underlying its entry into the respiratory tract epithelial cells [3]. GI epithelial cells heavily express the angiotensin-converting enzyme 2 (ACE 2). ACE 2 is the target for the entry of the SARS-CoV-2 virus in the lungs [4]. This proposed direct entry and effect along with patient exposure to pharmacologic therapies known to increase the risk of GI bleeding pose real concern for the gastroenterologist and raise questions regarding mortality and for the overall incidence and management of upper GI bleeding in COVID-19 patients.

There is very limited literature available at present regarding upper GI bleeding in COVID-19 patients due to the novelty of the disease. Available studies vary in outcome measures and design, further limiting the ability to interpret results [1, 5–7]. Given the continued occurrence of infections caused by the SARS-CoV-2 virus, there is an urgent need for additional research to guide gastroenterologists in the evaluation and management of this novel and complex patient population. As such, we aim to further investigate and characterize upper GI bleeding in SARS-CoV-2 patients compared to SARS-CoV-2–negative controls with GI bleeding by evaluating overall incidence, mortality, and use of upper GI endoscopy.

## 2. Methods

### 2.1. Study Design and Population

This was a retrospective, electronically matched 1:2 case–control study that reviewed patients 18 years or older admitted to our hospital system with an upper gastrointestinal bleed between January 1, 2020, and December 31, 2021. Data was collected on patients with upper GI bleed (UGB), endoscopy, and SARS-CoV-2–positive testing. Exclusion criteria for the patients included (i) negative or lack of SARS-CoV-2 testing at the time of endoscopy, (ii) negative or lack of endoscopy for UGB, (iii) repeat patients with a second admission for UGB, and (iv) insufficient secondary data such as past medical history and laboratory work. Exclusion criteria for the control group included insufficient secondary data and age under 18. These were randomly chosen during the above period based on gender and age, in a 1:2 case-to-control ratio. Patients were tested for the SARS-CoV-2 virus using a test based on real-time reverse transcription polymerase chain reaction. GI bleeding was defined as those undergoing endoscopy with signs of bleeding.

### 2.2. Data Collection

Test and control subjects were compared with respect to prior medical history, etiology of UGB, therapeutic intervention, laboratory findings, and in-hospital mortality. GI bleeding was characterized by the source of bleed (such as gastric ulcer, hemorrhagic gastritis, and Mallory–Weiss tear) and therapeutic interventions (including clip, cautery, injection, and medical interventions). Additionally, data were collected with respect to whether a repeat endoscopy was performed as well as for in-hospital mortality and cause of death. Other demographic information was collected including prior medical history, presentation/symptoms, labs (hemoglobin, platelet count, BUN, etc.), imaging results, and inpatient and outpatient medications. Outcome measures obtained include overall mortality and the use of GI endoscopy. The protocol for the research project was approved by our health system's Institutional Review Board. Data were collected using the hospital's electronic medical record system (EPIC).

### 2.3. Statistical Analysis

Descriptive statistics were reported as means (SD) and frequencies (percentages). All tests were two-tailed with a significance level of alpha = 0.05. Continuous variables were summarized using means and standard deviations. Categorical variables were expressed as percentages. All analyses were performed with SPSS 27 (IBM, Armonk, New York). The data reported are dichotomous/categorical, and chi-square testing was used for statistical analysis. Categorical/dichotomous variables are not normally distributed.

## 3. Results

**Figure 1:**
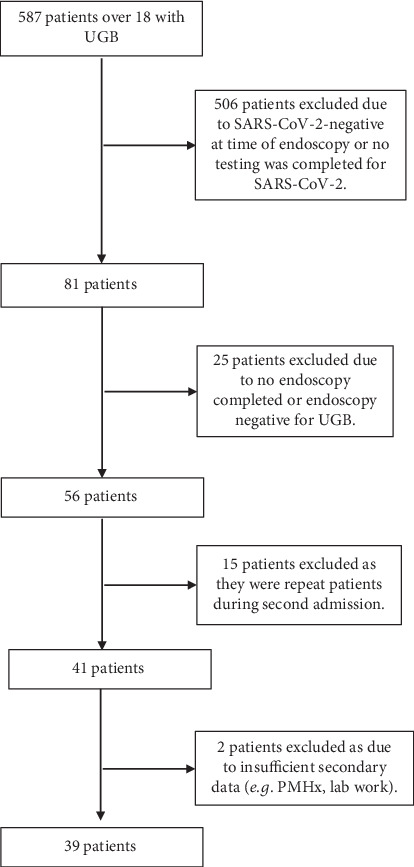
Overall scheme of the research project.

Data was collected for patients with UGB, endoscopy, and SARS-CoV-2–positive testing (*n* = 587) (Figure 1, flowchart). Five hundred and six patients were excluded due to SARS-CoV-2 testing negative at time of endoscopy or if no testing was completed for these patients. Twenty-five patients were excluded due to negative endoscopy for UGB or if no endoscopy was performed for these patients. Fifteen more patients were excluded as they were repeat patients with a second admission for UGB. Two patients were excluded due to insufficient secondary data such as past medical history and laboratory work. Test subjects (*n* = 39) included hospitalized patients with upper GI bleeding and tested positive for SARS-CoV-2 at the time of endoscopy, majority symptomatic. The mean age for SARS-CoV-2–positive and SARS-CoV-2–negative patients was 63.85 and 64.17 years, respectively. This age group has certain comorbidities. A univariate test comparing age (age < 50 years vs. age > = 50 years) to each of the comorbidities showed that these two age groups have significant difference for hypertension (*p* = 0.001), diabetes (*p* = 0.029), chronic kidney disease (*p* = 0.011), and cardiovascular disease (*p* = 0.001) (Supporting Information 1: Table S1). SARS-CoV-2–positive patients with GI bleeding had a higher rate of inpatient mortality compared to SARS-CoV-2–negative patients. Statistically significant differences observed between the SARS-CoV-2–positive and SARS-CoV-2–negative patients with respect to symptoms, medications, procedures, and laboratory values are summarized in Table 1. Duodenal ulcer, gastric ulcer, esophagitis, and erosive gastritis were the most common etiologies for GI bleeding in SARS-CoV-2–positive patients. These patients had significant progressive anemia and required more blood transfusions, steroids, proton pump inhibitors, and immunosuppressants.

Patients positive for SARS-CoV-2 were more likely to have leading presenting symptoms of severe progressive anemia (*p* < 0.001), shortness of breath (*p* < 0.001), fever (*p* = 0.006), cough (*p* = 0.002), abdominal pain (*p* = 0.041), and myalgia/fatigue (*p* = 0.029) than the patients negative for SARS-CoV-2 (Table 2). SARS-CoV-2–positive patients had a lower percentage of esophagitis compared to the SARS-CoV-2–negative group. SARS-CoV-2–positive patients had a higher percentage of duodenal ulcers compared to the control group. The SARS-CoV-2–positive and SARS-CoV-2–negative groups did not show significant differences with respect to the incidence of gastric ulcer, erosive or hemorrhagic gastritis, esophageal ulcer, gastric antral vascular ectasia (GAVE), and time from bleeding to endoscopy (Table 3). We also analyzed the Forrest classifications of ulcers (IB, IIA, IIB, IIC, III) between the control group and SARS-CoV-2–positive patients. Both groups had the highest percentage of ulcers ranked at III classification. SARS-CoV-2–positive patients had the smallest percentage of IIC, IIB classifications of ulcer. Almost one-quarter of both SARS-CoV-2–positive and SARS-CoV-2–negative patients had an endoscopy over 48 h after indications of bleeding.

Upon hospital admission, the SARS-CoV-2–positive patients had a higher respiratory rate, with a lower percentage of oxygen saturation (Supporting Information 2: Table S2). SARS-CoV-2–positive patients had a higher percentage of hematocrit and lower platelet count, compared to SARS-CoV-2–negative patients. A quarter of SARS-CoV-2–positive patients had a nasogastric (NG) tube placed prior to bleeding, while only a small fraction of SARS-CoV-2–negative patients had an NG tube placement (*p* = 0.003). There was a statistically significant difference (*p* = 0.031) in the use of room air between the SARS-CoV-2–positive and SARS-CoV-2–negative patients with majority of SARS-CoV-2–negative patients on room air during their hospital stay, indicating they did not need greater oxygen support. Furthermore, the SARS-CoV-2–positive group required more therapeutic interventions compared to the SARS-CoV-2–negative group. Of note, both groups were similar with respect to the in-hospital rebleeding, surgical intervention, and repeat endoscopy. In-hospital mortality was greater in SARS-CoV-2–positive patients than in SARS-CoV-2–negative patients (Supporting Information 3: Table S3). As for inpatient medications used for therapeutic management, SARS-CoV-2–positive patients were administered a higher percentage of PPIs (*p* = 0.03), steroids (*p* = 0.003), immunosuppressants (*p* = 0.002), aspirin (*p* = 0.03), enoxaparin therapeutic (*p* = 0.035) and prophylactic (*p* = 0.051), and heparin therapeutic and prophylactic, as well as directly acting oral anticoagulants/novel oral anticoagulants (DOAC/NOAC) (Table 4).

## 4. Discussion

We observed that there is a greater risk of mortality in patients with SARS-CoV-2 infection and GI bleeding than in those bleeding without the SARS-CoV-2 infection. Furthermore, the SARS-CoV-2–positive patients had significant progressive anemia and had to be given more therapeutic interventions such as blood transfusions, steroids, proton pump inhibitors, immunosuppressants, and ventilation compared to the SARS-CoV-2–negative patients. Based on our data, it is important for healthcare providers to be cognizant of the greater therapeutic needs of SARS-CoV-2–positive patients with UGB.

A matched case–control (1:2) study in patients with SARS-CoV-2 with and without GI bleeding that analyzed etiologies of bleeding and therapeutic approaches to treating patients with SARS-CoV-2 found that the most common UGIB etiologies were gastric or duodenal ulcers, similar to our observation [1]. A meta-analysis of 10 studies reported that patients with SARS-CoV-2 were at an increased risk of GI bleeding, particularly upper GI bleeding [8]. Another study that retrospectively evaluated bleeding incidence suggested that a more conservative approach to management is reasonable due to similar mortality and rebleeding rates when comparing aggressive treatment to conservative treatment [5]. Another retrospective study that looked at SARS-CoV-2–positive and SARS-CoV-2–negative patients with GI bleeding found a decreased survival rate in patients who underwent endoscopic evaluation regardless of COVID-19 status [6]. The authors suggested that the COVID-19 pandemic era in general is the cause of this finding and emphasized the impact this pandemic has had on the healthcare system. Moreover, a meta-analysis on acute GI bleeding in the COVID-19 patient reported peptic ulcer disease (PUD) to be the most common cause of bleeding. It also concluded that the overall mortality rate was high in patients with GI bleeding and SARS-CoV-2 infection but stressed the significant limitations and mixed results of the current outcome and management data available for interpretation in the clinical setting [7]. While the pathogenesis is not completely understood, a high prevalence of PUD complicated with bleeding was found to be associated with SARS-CoV-2–induced ARDS [5, 9]. Several mechanisms may contribute to the pathology of PUD and SARS-CoV-2, including epithelial damage caused by SARS-CoV-2, stress due to illness, or mucosal inflammation due to cytokine storm [9]. Similarly, our study found duodenal ulcers to be the most frequent cause of upper GI bleeding in SARS-CoV-2–positive patients, while esophagitis was the most frequent cause of upper GI bleeding in SARS-CoV-2–negative patients.

In our study, we focused on the risk of GI bleeding in SARS-CoV-2–positive patients. However, it is important to consider all complications related to SARS-CoV-2 infections for better treatment outcomes. A prospective study analyzed the prevalence and outcomes associated with hemorrhage, disseminated intravascular coagulopathy, and thrombosis (HECTOR) complicates in ICU patients with SARS-CoV-2 infection [10]. HECTOR complications occurred in 1732 of 11,929 patients studied; hemorrhagic complications occurred in 579 patients, most notably gastrointestinal hemorrhage making up 276 (48%) of hemorrhagic cases. Patients receiving extracorporeal membrane oxygenation (ECMO) were at a particularly increased risk of hemorrhagic complications. The study found an increased mortality with hemorrhagic complications, though no associated increase in mortality with thrombotic complications [10]. Not only is GI bleeding a vital risk to consider when treating patients, but thrombosis including pulmonary embolism, myocardial ischemia, deep vein thrombosis, and ischemic strokes should be important considerations for the management of SARS-CoV-2–positive patients.

There are many scoring systems developed to predict endoscopic intervention, mortality, and rebleeding in patients with UGB, such as the ABC score, Glasgow–Blatchford bleeding score, MAP, and the Japanese score [11]. Sex, age, hematemesis, and blood urea nitrogen were found to be predictors of the need for endoscopic intervention based on a retrospective study based in Korea [11]. We observed that nearly one-quarter of all patients had their first endoscopy over 48 h after bleeding was found. Endoscopies can localize the source of bleed and provide therapeutic treatment to stop the hemorrhaging. There is conflicting evidence regarding the time to endoscopy and decreased outcomes. A randomized controlled trial of 13 hospitals and 9009 patients with UGB reported that patients who had an endoscopy within 24 h had shorter lengths of hospital stay and a reduction in recurrent bleeding [12]. On the other hand, several studies analyzed patients with acute upper GI bleeding and their risk of 30-day mortality or risk of rebleeding based on the timing of endoscopy. Another randomized controlled trial with 598 patients did not find an association between 30-day mortality and rebleed in those receiving an endoscopy before 6 h as opposed to those receiving an endoscopy between 6 and 24 h [13]. There are several reasons why the patients in our study had a wait time of more than 48 h for endoscopy such as allowing the patients to be hemodynamically stable and following strict hospital protocols for SARS-CoV-2–positive patients [14]. While there is mixed evidence on the improvement of outcomes in endoscopy before 24 h, there is little evidence with respect to endoscopies after 48 h and it is recommended that the extensive wait time should be evaluated for impact on the patient treatment.

Though mirroring the same sizes of several other studies in the literature, our sample size was limited due to the difficulty of isolating patients with GI bleeding and SARS-CoV-2 positivity concurrently. In addition, the treatment of SARS-CoV-2 and other disease etiologies contribute to GI bleeding and provide difficulty in sorting out the primary cause of bleeding. However, there is no clear-cut method for determining the primary versus secondary cause of bleeding clinically. While PUD is the most common cause of GIB globally, it is interesting that the control group's most frequent cause of UGIB was esophagitis. To the best of our knowledge, there are no reports of esophagitis being more common during the pandemic. The current clinical practice worldwide may not routinely include testing for COVID-19 presence at present. Under these circumstances, it is practical to assume that patients with massive bleeding may be SARS-CoV-2 positive.

## 5. Conclusions

Given the ongoing nature of the pandemic, there is a continued need for additional research to guide gastroenterologists in the evaluation and management of complex patient populations. Our findings suggest that SARS-CoV-2–positive patients may require more intense management and monitoring for GI bleeding than SARS-CoV-2–negative patients. Our data helps shed light on the relationship between SARS-CoV-2 and GI bleeding due to SARS-CoV-2–related tissue damage and treatment affecting the GI tract.

## Figures and Tables

**Table 1:** Demographics and past medical history of the SARS-CoV-2-negative and SARS-CoV-2-positive patients.

**Demographics/prior medical history**	**COVID-19 positive**	**COVID-19 negative**	**p** ** value**
	**n** = 39	**n** = 78	
Sex			
Male	20 (51.3%)	40 (51.3%)	
Female	19 (48.7%)	38 (48.7%)	
Age—mean (std)			0.913
18–30	0 (0.0%)	1 (1.28%)	
31–40	3 (7.7%)	8 (10.3%)	
41–50	0 (0.0%)	10 (12.8%)	
51–60	7 (17.9%)	11 (14.1%)	
61–70	16 (41.0%)	17 (21.8%)	
71+	13 (33.3%)	31 (39.7%)	
Hypertension	29 (74.4%)	52 (67.9%)	0.475
Diabetes	21 (53.8%)	33 (42.3%)	0.238
Chronic kidney disease	9 (23.1%)	33 (42.3%)	0.236
Cardiovascular disease	14 (35.9%)	28 (35.9%)	1
Congestive heart failure	9 (23.1%)	10 (12.8%)	0.156
COPD/asthma	7 (17.9%)	18 (23.1%)	0.524
VTE	5 (12.8%)	13 (16.7%)	0.587
Active oncologic disease	7 (17.9%)	15 (19.2%)	0.867
Chronic liver disease	9 (23.1%)	11 (14.1%)	0.224
Neurologic disease	16 (41.0%)	22 (28.5%)	0.789
History of GI bleed	6 (15.4%)	22 (28.5%)	0.00125
Tobacco			0.759
Never	18 (46.2%)	39 (50%)	
Former	12 (30.8%)	19 (24.4%)	
Current	9 (23.1%)	20 (25.6%)	
Alcohol			0.152
Never	23 (59.0%)	41 (52.6%)	
Former	1 (2.6%)	11 (14.1%)	
Current	15 (38.5%)	26 (33.3%)	

**Table 2:** Symptoms reported by patients at time of admission.

**Symptoms**	**COVID positive**	**COVID negative**	**p** ** value**
	**n** = 39** (%)**	**n** = 78** (%)**	
Shortness of breath	23 (59.0%)	8 (10.3%)	< 0.001
Fever	7 (17.9%)	2 (2.6%)	0.006
Cough	7 (17.9%)	1 (1.3%)	0.002
Abdominal pain	9 (23.1%)	33 (42.3%)	0.041
Myalgia/fatigues	16 (41.0%)	17 (21.8%)	0.029
Diarrhea	8 (20.5%)	12 (15.4%)	0.487
Melena	26 (66.7%)	52 (66.7%)	1
Hematochezia	4 (10.3%)	8 (10.3%)	1
Severe progressive anemia	36 (92.3%)	47 (60.3%)	< 0.001
Coffee ground emesis	6 (15.4%)	13 (16.7%)	0.859
Maroon-colored stool	3 (7.7%)	3 (3.8%)	0.399
Hematemesis	6 (15.4%)	20 (25.6%)	0.208

**Table 3:** Bleeding etiologies found on endoscopy.

**Endoscopy findings**	**COVID positive**	**COVID negative**	**p** ** value**
	**n** = 39** (%)**	**n** = 78** (%)**	
Esophagitis	3 (7.7%)	14 (17.9%)	0.138
Duodenal ulcer	12 (30.8%)	12 (15.4%)	0.052
Gastric ulcer	3 (7.7%)	10 (12.8%)	0.405
Erosive or hemorrhagic gastritis	3 (7.7%)	9 (11.5%)	0.748
Malignant lesion	0 (0.0%)	2 (2.6%)	0.552
Gastroesophageal varices	4 (10.3%)	5 (6.4%)	0.479
Esophageal ulcer	2 (5.10%)	3 (3.8%)	1
Angioectasis	4 (10.3%)	2 (2.6%)	0.094
Marginal ulcer	2 (5.10%)	0 (0.0%)	0.3
GAVE	1 (2.6%)	1 (1.3%)	1
Cameron lesion	0 (0.0%)	3 (3.8%)	0.55
Other	2 (5.10%)	8 (10.3%)	0.492
Time from bleeding concern to endoscopy			0.897
0–12 h	9 (23.1%)	15 (19.2%)	
12–24 h	6 (15.4%)	18 (23.1%)	
24–36 h	9 (23.1%)	17 (21.8%)	
36–48	6 (15.4%)	10 (12.8%)	
> 48 h	9 (23.1%)	18 (23.1%)	

*Note:* Time from bleeding to endoscopy: time when bleeding was identified to time endoscopy was performed.

**Table 4:** Inpatient and outpatient medications.

	**COVID positive**	**COVID negative**	**p** ** value**
	**n** = 39	**n** = 78	
Inpatient medications			
H2 blocker daily	7 (17.9%)	9 (11.5%)	0.340
H2 blocker twice daily	4 (10.3%)	2 (2.6%)	0.094
PPI daily	8 (20.5%)	5 (6.4%)	0.030
PPI twice daily	20 (51.3%)	32 (41%)	0.293
PPI infusion	19 (48.7%)	54 (69.2%)	0.031
NSAID	1 (2.6%)	0 (0%)	0.333
Steroid	10 (25.6%)	5 (6.4%)	0.003
Immunosuppressant	9 (23.1%)	3 (3.8%)	0.002
Aspirin alone	25.60%	10.30%	0.030
Clopidogrel alone	1 (2.6%)	1 (1.3%)	1.000
Dual antiplatelet therapy	1 (2.6%)	2 (2.6%)	1.000
Enoxaparin therapeutic	3 (7.7%)	0 (0%)	0.035
Enoxaparin prophylactic	10 (25.6%)	9 (11.5%)	0.051
Heparin therapeutic	7 (17.9%)	6 (7.7%)	0.121
Heparin prophylactic	8 (20.5%)	7 (9%)	0.078
Warfarin	0 (0.0%)	1 (1.3%)	1.000
DOAC/NOAC	9 (23.1%)	3 (3.8%)	0.399
Outpatient medications			
H2 blocker daily	2 (5.1%)	4 (5.1%)	1.000
H2 blocker twice daily	1 (2.6%)	0 (0%)	0.333
PPI daily	9 (23.1%)	22 (28.2%)	0.553
PPI twice daily	3 (7.7%)	10 (12.8%)	0.540
NSAID	6 (15.4%)	7 (9.0%)	0.354
Steroid	4 (10.3%)	3 (3.8%)	0.220
Immunosuppressant	2 (5.1%)	5 (6.4%)	1.000
Aspirin alone	5 (12.8%)	21 (26.99%)	0.202
Clopidogrel alone	2 (5.1%)	4 (5.1%)	1.000
Dual antiplatelet therapy	4 (10.3%)	10 (12.8%)	0.687
Enoxaparin therapeutic	0 (0.0%)	2 (2.6%)	0.552
Enoxaparin prophylactic	0 (0.0%)	1 (1.3%)	1.000
Warfarin	1 (2.6%)	4 (5.1%)	0.664
DOAC/NOAC	3 (7.7%)	13 (16.7%)	0.183

## Data Availability

The data that support the findings of this study are available on request from the corresponding author. The data are not publicly available due to privacy or ethical restrictions.

## Supporting Information

Supporting Information 1 Table S1: Age as a factor with respect to comorbidities.

Supporting Information 2 Table S2: Physical exam and laboratory data.

Supporting Information 3 Table S3: Hospital stay data.
