# Supplementary material for: Incidence and Outcomes of Upper GI Bleeding in Hospitalized SARS-CoV-2 Patients
Source: Gastroenterol Res Pract. 2025 Mar 31;2025:4358786. doi: 10.1155/grp/4358786 (PMC11976044; doi:10.1155/grp/4358786)
Supplement: Supporting Information 2 — Table S2: Physical exam and laboratory data. [file 4358786.f2.docx]

**Supplementary Table 2: Physical exam and laboratory data**

Lab values were the first set of values from hospital admission.
