# Supplementary material for: Incidence and Outcomes of Upper GI Bleeding in Hospitalized SARS-CoV-2 Patients
Source: Gastroenterol Res Pract. 2025 Mar 31;2025:4358786. doi: 10.1155/grp/4358786 (PMC11976044; doi:10.1155/grp/4358786)
Supplement: Supporting Information 3 — Table S3: Hospital stay data. [file 4358786.f3.docx]

**Supplementary Table 3: Hospital stay data**

† Therapeutic intervention: any intervention except clip, cautery, injection
